# Painful diabetic neuropathy is associated with accelerated epigenetic aging

**DOI:** 10.1007/s11357-025-01516-w

**Published:** 2025-01-23

**Authors:** Katarzyna Malgorzata Kwiatkowska, Paolo Garagnani, Massimiliano Bonafé, Maria Giulia Bacalini, Luciano Calzari, Davide Gentilini, Dan Ziegler, Monique M. Gerrits, Catharina G. Faber, Rayaz A. Malik, Margherita Marchi, Erika Salvi, Giuseppe Lauria, Chiara Pirazzini

**Affiliations:** 1 Department of Medical and Surgical Sciences (DIMEC), University of Bologna, Bologna, Italy; 2 IRCCS Azienda Ospedaliero-Universitaria Di Bologna, Bologna, Italy; 3 Department of Biomedical and Neuromotor Sciences (DIBINEM), University of Bologna, Bologna, Italy; 4 Bioinformatics and Statistical Genomics Unit, Istituto Auxologico Italiano IRCCS, Cusano Milanino, Italy; 5 Department of Brain and Behavioral Sciences, University of Pavia, Pavia, Italy; 6 Institute for Clinical Diabetology, German Diabetes Center, Leibniz Center for Diabetes Research at Heinrich Heine University, Düsseldorf, Germany; 7 Department of Clinical Genetics, Maastricht University Medical Centre+, Maastricht, Netherlands; 8 Department of Neurology, Institute of Mental Health and Neuroscience, Maastricht University Medical Centre+, Maastricht, Netherlands; 9 Institute of Cardiovascular Sciences, Manchester University NHS Foundation Trust, Manchester Academic Health Science Centre, Manchester, England; 10 Weill Cornell Medicine-Qatar, Ar-Rayyan, Doha Qatar; 11 Department of Clinical Neurosciences, Neuroalgology Unit, Fondazione IRCCS Istituto Neurologico “Carlo Besta”, Milan, Italy; 12 Department of Medical Biotechnology and Translational Medicine, University of Milan, Milan, Italy

**Keywords:** Biological aging, Aging biomarker, Epigenetic clock, Neuropathic pain, Diabetic neuropathy, DNA methylation

## Abstract

About one out of two diabetic patients develop diabetic neuropathy (DN), of these 20% experience neuropathic pain (NP) leading to individual, social, and health-economic burden. Risk factors for NP are largely unknown; however, premature aging was recently associated with several chronic pain disorders. DNA methylation-based biological age (DNAm) is associated with disease risk, morbidity, and mortality in different clinical settings. The purpose of this work was to study, for the first time, whether biological age is involved in pain development in a huge cohort of DN patients with neuropathy assessed by anatomopathological assay (99 painful (PDN), 132 painless (PLDN) patients, 84 controls (CTRL)). Six subsets of DNAm biomarkers were calculated to evaluate NP-associated changes in epigenetic aging, telomere shortening, blood cell count estimates, and plasma protein surrogates. We observed pain-related acceleration of epigenetic age (DNAmAgeHannum, DNAmGrimAgeBasedOnPredictedAge, DNAmAgeSkinBloodClock), pace of aging (DunedinPoAm), and shortening of telomeres between PDN and PLDN patients. PDN showed decreased predicted counts of B lymphocytes, naive and absolute CD8 T cells, and increased granulocyte counts. Several surrogates of plasma proteins were significantly different (GHR, MMP1, THBS2, PAPPA, TGF-α, GDF8, EDA, MPL, CCL21) in PDNs compared to PLDNs. These results provide the first evidence of an acceleration of biological aging in patients with painful compared to painless DN. This achievement has been possible thanks to the state of the art clinical phenotyping of the enrolled patients. Our findings indicate that the aging process may be directly involved in the PDN progression and in general health degeneration in the T2DM patients. Therefore, it is possible to hypothesize that the administration of effective antiaging drugs could slow down or even block the disease advancement.

**Supplementary Information:**

The online version contains supplementary material available at 10.1007/s11357-025-01516-w.

## Introduction

Neuropathic pain (NP) is caused by a lesion or disease of the somatosensory nervous system [1] and is distinguished from other types of pain by simultaneous sensory loss and pain, with or without sensory hypersensitivity with allodynia or hyperalgesia [2]. NP often affects patients with acquired peripheral neuropathies—a group of disorders characterized by the degeneration of sensory and motor nerve fibers due to systemic diseases like diabetes or chemotherapy [3, 4]. These pathological changes lead to somatosensory pathway impairment with loss of perception to stimuli such as touch, pressure, temperature, and nociception mainly in the feet. Moreover, sensory nerve damage causes spontaneous positive symptoms, such as paresthesia, tingling, burning pain, sensation of tightness or paroxysmal shooting, and allodynia, namely painful sensations caused by non-painful stimuli.

The diagnosis of peripheral neuropathy is achieved by bedside clinical examination, nerve conduction studies, and skin biopsy. Due to its high prevalence, type 2 diabetes mellitus (T2DM) is the most common cause of painful neuropathy worldwide. However, despite a similar degree of peripheral nerve degeneration, only 20% of patients develop painful diabetic neuropathy (DN). The mechanisms underlying this variability remain unknown and cannot be explained by genetic alterations [5].

The pathological acceleration of biological aging has been directly correlated with chronological age, is linked to several age-related health conditions and complications, and can predict lifespan. Biological aging can be measured through a wide range of predictors including telomere length [6], the transcriptome of a specific set of genes [7, 8], glycan composition [9], and specific changes in the proteome [10], metabolome [11, 12], and methylome [13–17]. Moreover, structural neuroimaging allows brain-predicted aging estimation [18]. Some of these signatures, particularly those related to epigenetics, telomere, and neuroimaging changes, were previously reported to be altered in chronic pain disorders [19–23].

The purpose of the present work was to investigate epigenetic aging expressed by DNA methylation-based models in patients diagnosed with painful or painless DN, and sex- and age-matched healthy subjects.

## Methods

### Study cohorts

The two independent cohorts used for the experimental design, PROPGER and PROPENG, originated from the European funded project “Molecule-to-man pain network” (PAIN-Net, H2020-MSCA-ITN-2016, Grant Agreement number: 721841) focused on diabetic neuropathy. The PROPGER cohort was provided by the University of Maastricht (NL) and consisted of painful and painless DN individuals recruited by the German Diabetes Center in Düsseldorf (DE). Individuals were 93% of German ethnicity, 3% of non-German European ethnicity, 3% of Asian, 0.6% of African, and 0.3% mixed origin. The PROPENG cohort consisted of painful and painless DN subjects provided by Fondazione IRCCS Istituto Neurologico Carlo Besta in Milan (IT) and Manchester University (UK), and were 79% of European ethnicity, 18% of Asian, 2% of Afrocaribbean, and 1% mixed origin.

Additionally, a control group (CTRL) was recruited from healthy Italian subjects from a collection at the Department of Experimental, Diagnostic and Specialty Medicine (DIMES) of the University of Bologna (IT). All study participants gave their signed informed consent.

All patients underwent clinical examination and phenotype characterization within the PAIN-Net consortium uniform diagnostic approach and shared protocols to guarantee homogeneous subgroup clustering. Neuropathy was determined from the result of skin biopsy [24] and patients were considered as “painful” if they experienced neuropathic pain for more than 1 year and if their pain intensity reached a Numeric Rating Scale NRS of 4 or greater [25].

### Sample selection

The population genomic background is heterogeneous being shaped by demographic history, neutral and adaptive evolution, and their complex interplay [26]. Genetic variations are closely linked to DNA methylation and can easily influence the epigenetic patterns, introducing confounding variability in the data that should not be neglected [27]. Therefore, in order to reduce the bias coming from population genetics, we included exclusively individuals of European ethnicity. Type 1 and 2 diabetes induces different biological changes influenced by differing genetic predisposition [28], which could be a source of additional variability. Thus, only T2DM subjects were selected. Whenever possible, it was attempted to match painful, painless, and control samples regarding the chronological age and sex since both factors were shown to alter the DNA methylation levels [29, 30].

### DNA extraction and methylation experiment

DNA was extracted from whole blood of DN patients and healthy controls using respectively Puregene Blood kit (Qiagen) and QIAamp DNA Blood Mini kit (Qiagen), following the steps of corresponding protocols. The use of different DNA extraction kits is unlikely to have a significant effect on observed differences between the groups, and it should not falsify or distort the detected methylation patterns associated with phenotype of interest [31]. All the samples were quantified with Qubit dsDNA Broad Range Assay kit on Qubit Fluorometer by Thermo Fisher Scientific and verified to contain ≥ 1000 ng of DNA, i.e. the amount required for a methylation assay. A total of 1000 ng of genomic DNA was normalized in 50µL of H_2_O and were used in bisulfite conversion with EZ DNA Methylation Kit (Zymo Research) following the manufacturer’s instructions. Genome-wide DNA methylation experiment was performed with Infinium HumanMethylationEPIC BeadChip (Illumina) according to original protocol. Within each array the samples and phenotypic groups were accurately randomized.

### Preprocessing of raw data

All the handling and manipulation of files were performed in Linux environment. Illumina output data (files in*.idat* format) from the experiments were parsed and preprocessed using *minfi* package within R software (version 3.6.3). Low-quality samples with mean probe detection *p*-value above 0.05 were excluded from the analysis and the probes that failed (i.e. presented detection *p*-value > 0.01) in at least one of the samples were removed. The raw values of intensities in green and red channels were normalized using functional approach removing undesired variation with a regression model of explained variability based on the control probes included in the array (using *preprocessQuantile* function from *minfi* package). Beta values estimating the methylation levels as a ratio of methylated to unmethylated alleles intensities, ranging between 0 (totally unmethylated) and 1 (totally methylated), were calculated for all the samples and used in subsequent biological age estimation.

### Estimation of DNAm-based biomarkers

We assessed six subsets of biomarkers based on DNA methylation: (a) epigenetic clocks and the component parts of DNAmGrimAge clock, (b) bolstered clock models (PC-Clocks), (c) surrogates of blood cell counts, (d) predictions of biological traits, (e) signature of chronic low-grade inflammation as measured by serum levels of C-reactive protein (CRP-associated risk score), and (f) plasma protein surrogates (EpiScores). List of all calculated DNAm-based variables with the corresponding references and computation methods used is provided in Supplementary TableS1. Respective assessments were performed online or implemented in R (v4.2.2) or Python (v3.9.16) environments installed on Linux OS using normalized methylation Beta values as an input. Eventual outliers (samples for which DNAm-based biomarkers were below Q1–1.5IQR or above Q3 + 1.5IQR, where Q1 is the first quartile, Q3 is the third quartile, and IQR is the interquartile range) were removed prior to two-stage residual-outcome regression analysis.

### Two-stage residual-outcome regression model

The variations in DNAm-based estimates in studied phenotypes were investigated applying a two-stage residual-outcome regression approach. Particularly, for each biomarker, we built a reference linear regression model *lm(y* ~ *x)* with the calculated estimate as dependent variable *y*, with chronological age as independent variable *x* whilst correcting for sex covariate, using in the fitting step exclusively data from the subjects with PLDN. This generated model was used to predict the respective value of epigenetic variables in the PDN and CTRL groups, and to subsequently calculate the residuals—*i.e.* the distance between experimental values and modeled regression line—corrected for chronological age. The different phenotypic groups were compared using Student’s *t*-test on age-adjusted residuals with a 0.05 statistical significance level.

## Results

We analyzed T2DM patients with painful or painless DN and a group of healthy individuals as controls. For all the groups, six subsets of DNAm-based biomarkers were estimated, including methylation age measures according to different available epigenetic clocks with the component parts of GrimAge clock and telomere length estimator, PC bolstered models of classic clocks, surrogates of blood cell counts, predictions of biological traits, CRP-associated risk score, and EpiScores of plasma protein surrogates. The differences in the parameters among phenotypic groups within studied populations were examined with two-stage residual-outcome regression approach.

### Cohort profiles

After removal of low-quality samples, there were 99 PDN cases (mean age 67 years; age range 46–83), 132 PLDN patients (mean age 67 years; age range 41–84), and 84 CTRL subjects (mean age 64 years; age range 41–78) as summarized in Table 1. Groups did not differ significantly in mean age (*p*-value from ANOVA test = 0.051). The average duration of T2DM was 13 years (ranging between 0 and 46) and 11 years (ranging between 0 and 44) in PDN and PLDN patients, respectively. Although the PDN patients had a longer duration of diabetes than the PLDN patients, this difference was not statistically significant (*p*-value from Student’s two-sided *t*-test for samples with equal variances = 0.080).

**Table 1:** Characteristics of studied cohort. Sample size, sex distribution, mean age, and duration of T2DM for each phenotypic group of studied cohorts are reported. *PDN* painful diabetic neuropathy, *PLDN* painless diabetic neuropathy, *CTRL* controls, *T2DM* type 2 diabetes mellitus, *SD* standard deviation, *NA* not applicable

	**PDN**	**PLDN**	**CTRL**
**Number of samples** (%)	99 (31.4%)	132 (41.9%)	84 (26.7%)
**Sex** (females/males)	24/75	33/99	26/58
**Age average** ± SD (years)	67.0 ± 9.5	67.3 ± 9.3	64.3 ± 9.0
**T2DM duration** (years)	13.5 ± 10.1	11.3 ± 8.5	NA

### NP-related DNAm-based biomarkers

DNAm-based estimates were analyzed with a two-stage residual-outcome regression approach in 99 PDN and 132 PLDN patients, and 84 healthy controls to identify the DNA methylation-based changes in epigenetic aging and in other biological surrogates linked to PDN.

**Fig. 1:**
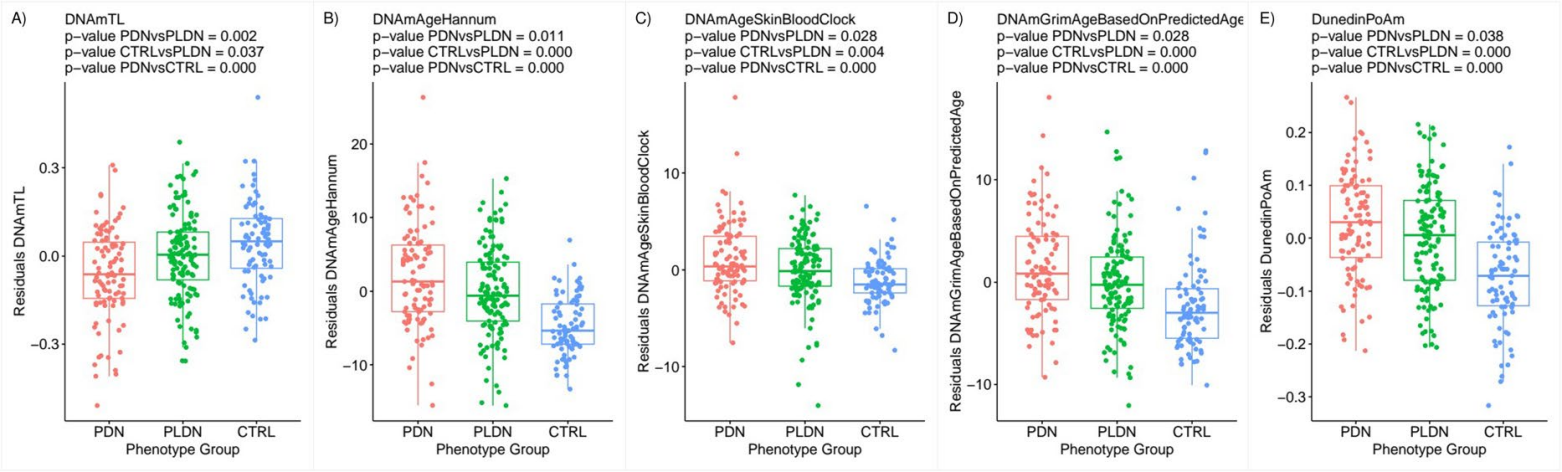
Alterations in epigenetic biomarkers expressed by **A** DNAmTL, **B** DNAmAgeHannum, **C** DNAmAgeSkinBloodClock, **D** DNAmGrimAgeBasedOnPredictedAge, and **E** DunedInPoAm models, observed in PDN, PLDN, and CTRL groups (*X* axis). Residuals from the two-stage residual-outcome regression approach with the PLDN group as the reference fit are reported on the *Y* axis. For each phenotype contrast, *p*-values from Student’s *t*-test are disclosed

Analysis of DNAm biomarkers from *subset A* demonstrated significant pain-related accelerated epigenetic aging in the PDN compared to PLDN group. Details of results from two-stage residual-outcome regression are provided in Supplementary Table S2. In particular, telomere shortening, a well-known hallmark of both cellular senescence and biological aging, estimated with DNAmTL model in this work, was significantly increased (*p*-value = 0.002) in PDN as illustrated in Fig. 1A. There was an acceleration of biological age expressed by DNAmAgeHannum (*p*-value = 0.011) and DNAmAgeSkinBloodClock (*p*-value = 0.028) clocks. Although the predictor of DNAmGrimAgeBasedOnPredictedAge was increased (*p*-value = 0.028) in PDN patients, none of the separate components of the model reached statistical significance. Subjects with PDN also had an increased pace of aging (*p*-value = 0.038) calculated using DunedInPoAm algorithm. Figure 1B–E visualize residuals of estimated values of significant epigenetic clocks.

**Fig. 2:**
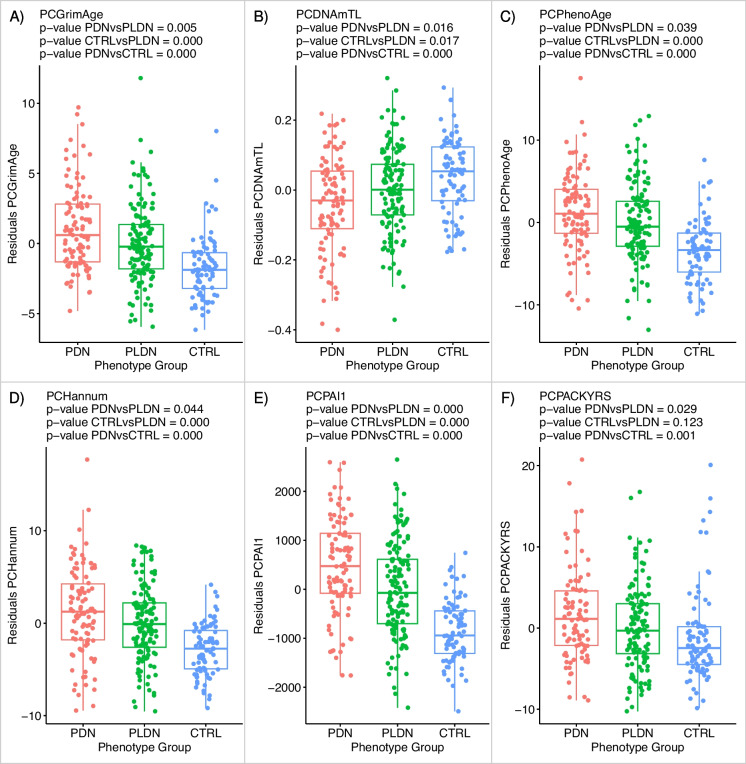
Alterations in epigenetic biomarkers expressed by **A** PC-DNAmGrimAge, **B** PC-DNAmTL, **C** PC-DNAmPhenoAge, **D** PC-DNAmHannum, **E** PC-PAI1, and **F** PC-PACKYRS models, observed in PDN, PLDN, and CTRL groups (*X* axis). Residuals from the two-stage residual-outcome regression approach with the PLDN group as the reference fit are reported on the *Y* axis. For each phenotype contrast, *p*-values from Student’s *t*-test are disclosed

Biomarkers of *subset B* included models improved with principal component analysis of several standard predictors present in subset A: Horvath’s DNAmAge, DNAmAgeHannum, DNAmGrimAge and its components, DNAmPhenoAge, and DNAmTL. Results of the regression analysis are reported in Supplementary Table S3. The outcome of bolstered algorithms replicated a significant acceleration of epigenetic clocks in the PDN group for the following models: PC-DNAmGrimAge (*p*-value = 0.005), PC-DNAmTL (*p-*value = 0.016), PC-DNAmPhenoAge (*p*-value = 0.039), and PC-DNAmAgeHannum (*p*-value = 0.044) as shown in Fig. 2A–D. Additionally, using amended models, we found increased predicted plasma levels of plasminogen activator inhibitor antigen type 1 (PC-PAI1) and a higher predicted number of cigarette packs smoked/year (PC-PACKYRS) which are both component parts of DNAmGrimAge (Fig. 2E and 2F).

**Fig. 3:**
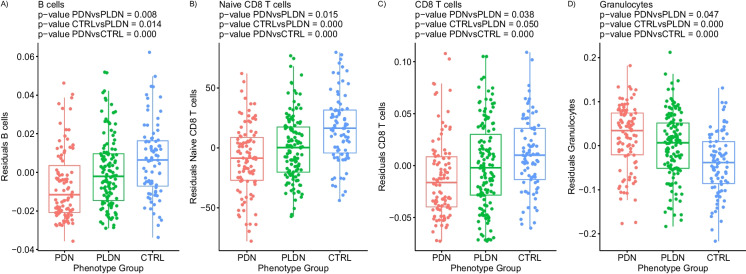
Alterations of DNAm-based blood cell counts estimates of **A** B lymphocytes, **B** naive CD8 T cells, **C** CD8 T cells, and **D** granulocytes, observed in PDN, PLDN, and CTRL groups (*X* axis). Residuals from the two-stage residual-outcome regression approach with the PLDN group as the reference fit are reported on the *Y* axis. For each phenotype contrast, *p*-values from Student’s *t*-test are disclosed

Our DNAm biomarker *subset C* included estimates of blood cell counts. Results of differential analysis are reported in Supplementary TableS4. B lymphocytes and naïve and absolute CD8 T cell surrogates were decreased in PDN compared to PDLN patients, with *p*-values of 0.008, 0.015, and 0.038, respectively. Moreover, a methylation-based predictor of the granulocyte count was significantly higher (*p*-value = 0.047) in PDN compared to PLDN patients. Pain-related alterations in estimates of blood cell counts are visualized on Fig. 3.

No significant differences between the two phenotypes were found for the *subset D* biomarkers including biological traits such as waist-to-hip ratio, HDL cholesterol levels, body fat levels, body mass index, and predicted alcohol and cigarette use (Supplementary Table S5). Similarly, analysis of *subset E* involving a signature of the risk score for chronic low-grade inflammation measured by serum levels of CRP did not differ between PDN and PLDN patients (Supplementary Table S6 ).

**Fig. 4:**
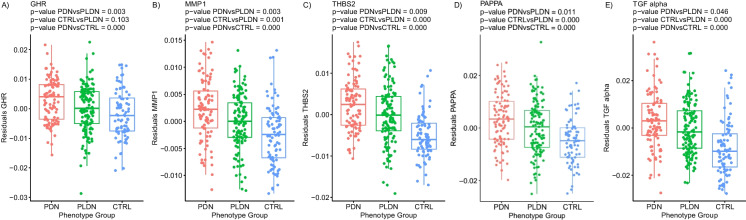
Alterations of DNAm-based plasma protein surrogates of **A** growth hormone receptor (GHR), **B** interstitial collagenase (MMP1), **C** thrombospondin-2 (THBS2), **D** pappalysin-1 (PAPPA), and **E** transforming growth factor alpha (TGF-α), observed in PDN, PLDN, and CTRL groups (*X* axis). Residuals from the two-stage residual-outcome regression approach with the PLDN group as the reference fit are reported on the *Y* axis. For each phenotype contrast, *p*-values from Student’s *t*-test are disclosed

**Fig. 5:**
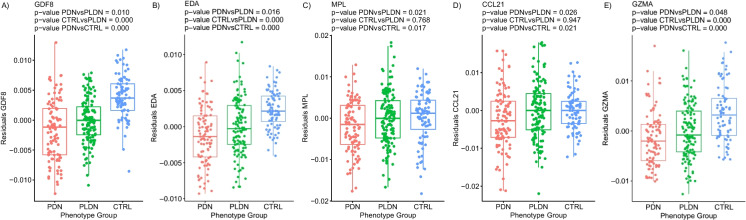
Alterations of DNAm-based plasma protein surrogates of **A** growth/differentiation factor 8 (GDF8), **B** ectodysplasin-A (EDA), **C** thrombopoietin receptor (MPL), **D** C–C motif chemokine 21 (6-Ckine; CCL21), and **E** granzyme A (GZMA), observed in PDN, PLDN, and CTRL groups (*X* axis). Residuals from the two-stage residual-outcome regression approach with the PLDN group as the reference fit are reported on the *Y* axis. For each phenotype contrast, *p*-values from Student’s *t*-test are disclosed

Finally, analysis of DNAm biomarker *subset F* of EpiScores which are plasma protein surrogates returned ten estimates that were related to pain. A comprehensive list of all EpiScore results is provided in Supplementary Table S7 . Predicted levels of growth hormone receptor (*GHR*; *p*-value 0.003), interstitial collagenase (*MMP1*; *p*-value 0.003), thrombospondin-2 (*THBS2*; *p*-value 0.009), pappalysin-1 (*PAPPA*; *p*-value 0.011), and transforming growth factor alpha (*TGF-α*; *p*-value = 0.046) proteins were significantly increased in patients with PDN compared to PLDN (Fig. 4). Plasma growth/differentiation factor 8 (*GDF8*; *p*-value 0.010), ectodysplasin-A (*EDA*; *p*-value 0.016), thrombopoietin receptor (*MPL*; *p*-value 0.021), C–C motif chemokine 21 (*6-Ckine*/*CCL21*; *p*-value 0.026), and granzyme A (*GZMA*; *p*-value = 0.048) levels were lower in the patients with PDN compared to PLDN (Fig. 5). We investigated possible correlations between the above parameters with biological ageing in order to uncover a direct influence of ageing speed on these results. Pearson’s correlation between significant DNAm-based clocks and EpiScores was analyzed (Supplementary Figures S1-S10). Even if there were several significant correlations, *r* coefficient ranged between 0 and 0.5 indicating weak to moderate strength of correlations (the highest correlation was found between *THBS2* and DNAmAgeHannum: *r* = 0.505 and *p*-value < 0.001).

Notably, all DNAm-based biomarkers revealed a consistent phenotype-related trend, where the PLDN group oscillated at the baseline levels since it was used as the reference group to build a regression model, and PDN and CTRL groups presented opposite shifts corresponding to alterations in epigenetic surrogates.

## Discussion

To the best of our knowledge, this is the first study evaluating epigenetic aging in a cohort of T2DM patients with painful and painless diabetic neuropathy. We provide evidence of significant differences in epigenetic clocks and in a battery of DNAm-based biomarkers between T2DM patients with painful and painless diabetic neuropathy.

We found consistent NP-related acceleration in DNAmAgeHannum, DNAmGrimAgeBasedOnPredictedAge, and DNAmAgeSkinBloodClock models, as well as in DunedinPoAm that captures individual variation in the pace of biological aging which was increased in PDN patients. Several studies previously found associations between epigenetic aging and chronic pain despite differences in experimental designs, cohort numbers, subject phenotype, and evaluation tools used. Acceleration of DNAmAge was observed in self-reported chronic pain in a cohort of healthy community-dwelling adults [20]. DunedinPACE was associated with chronic low back pain, its intensity and interference [19, 32]. DNAmGrimAge was positively correlated with self-reported knee pain [21, 23, 33] and knee osteoarthritis pain [22, 34].

DNAm-based prediction of telomere length further confirmed acceleration of epigenetic aging in patients with NP and indeed we observed an increased shortening of telomeres in the PDN group. DNA methylation-based estimation of telomere length is highly robust and provides a more accurate prediction of disease outcomes and all-cause mortality risk [35]. Our findings are consistent with a previous study indicating a relationship between chronic pain and reduced telomere length in women with fibromyalgia comparing to healthy controls [36]. Another study in patients with fibromyalgia showed a negative correlation between pain measured with the McGill Pain Questionnaire and DNAmTL [37]. A study in women with migraine and matched controls also showed increased telomere shortening [38]. Subjects with chronic knee osteoarthritis pain had significantly shorter telomeres than individuals without or with low pain intensity [39, 40].

Advanced epigenetic age in the PDN group was reproduced with principal-component-based versions of the standard models. PC-DNAmGrimAge, PC-DNAmPhenoAge, PC-DNAmAgeHannum confirmed the acceleration of methylation clocks, and PC-DNAmTL replicated increased shortening of telomeres. These alternative algorithms offer improved control of the estimation noise and enhanced reliability of predicted clocks [41, 42].

Amongst DNA methylation-based estimates of blood cell counts, we observed decreased surrogate values of B lymphocytes, naive and absolute CD8 T cells, and increased granulocyte counts in PDN patients compared to the PLDN group. During aging, the production of B and T lymphocytes drastically declines affecting efficiency of the immune system, increasing the risk of infections and autoimmune diseases [43, 44]. An increased estimated granulocyte count was found in patients with Parkinson’s disease and it correlated with intrinsic and extrinsic epigenetic age acceleration [45]. To our knowledge, this is the first study showing that alterations in blood cell counts are related to NP-related age acceleration demonstrated in the DNAm clock analysis.

The EWAS data also enabled the calculation of EpiScores to estimate relative levels of several plasma proteins, providing interesting insights. Analysis of plasma protein surrogates indicated a pain-related reduction in predicted levels of plasma C–C motif chemokine 21 (***CCL21***) in the PDN group. *CCL21*was shown to evoke hypersensitivity [46, 47], to contribute to neuropathic pain [48–50], and to play a role in inflammation and associated degeneration [51–53]. Our analysis demonstrated increased DNAm-based surrogate of growth hormone (***GHR***) in the PDN group. *GHR* was previously associated with fibromyalgia [54, 55], chronic pain [56–58], diabetes, and age-related pathologies including inflammatory disorders, stroke, and neurodegenerative diseases [59, 60]. In our study, DNAm-based predictor of plasma thrombospondin-2 (***THBS2***) was increased in the PDN group. Increased *THBS2* glycoprotein has recently been observed in diabetic nephrophaty patients [61] and in accelerated aging conditions [62, 63]. Estimates of pappalysin 1 (***PAPPA***) were higher in both PLDN and PDN patients. Protein *PAPPA* was shown to be associated with risk of diabetes [64], to contribute to the development and progression of age-related degenerative changes [65–67], and to promote the longevity in case of its deficiency [65, 68, 69]. DNAm-based surrogate of ***TGF****-α* was decreased in studied PDN patients. This protein was previously reported to be involved in the progression of diabetic neprophaty [70], in cognitive decline and neuropathological aging [71]. Growth differentiation factor 8 (***GDF8***), also known as myostatin, was significantly decreased in PDN patients. *GDF8* was previously negatively associated with diabetes [72] and it was proposed as a potential target for rejuvenation (not confirmed in experimental studies) [73]. In our cohort, low estimated plasma levels of granzyme A (***GZMA***) were associated with PDN. *GZMA* was previously reported in pediatric type 1 diabetes [74] and it was also recognized as a proinflammatory mediator contributing to overreaction of the immune system with a reduced inflammatory response [75–77]. Predicted levels of ectodysplasin A (***EDA***) were lower in the PDN compared to PLDN group. *EDA* hepatokine was found to be overexpressed in T2DM [78, 79] and to be involved in bone homeostasis and osteopetrosis-like skeletal changes [80–82]. EpiScore estimating plasma level of interstitial collagenase (***MMP1***) was significantly higher in the PDN compared to the PLDN group. *MMP1* was previously reported in painful joint pathologies [83], rheumatoid arthritis, and osteoarthritis [84–86]. EpiScore of thrombopoietin receptor (***MPL***) was lower in PDN compared to PLDN. *MPL* has been related to hematological disorders, where its deficiency led to thrombocytopenia and bone marrow failure [87–89] and its enhanced functioning drove the development of myeloproliferative neoplasms [90–92]. Analysis of correlation between DNAm-based clocks and EpiScores confirmed that altered plasma protein levels are linked to a phenotype rather than to an accelerated aging.

Interestingly, for a great part of the estimates, significant differences were found not only between PDN and PLDN phenotypes, but also between both PDN/PLDN and healthy controls. The three groups could reflect the evolving stages of diabetic neuropathy, where CTRL represents a biological state in which diabetic neuropathy may or may not occur, PLDN embodies the state of development and progression, and PDN corresponds to a severe form in which the pattern of DNA methylation-based estimates captures the biological aging associated with its progression.

We have also observed a large set of epigenetic variables that did not differ between PDN and PLDN, but that varied when compared between PDN/PLDN groups and controls. These findings indicate that traits characterizing DN, independently of the development of NP, must exist. This is not surprising, because DN patients presented with accelerated biological age expressed by Horvath’s DNAmAge, PhenoAge, and AltumAge models when compared to healthy subjects. Several previously published studies have shown a positive association between epigenetic clocks and diabetes [93–95] despite differences in experimental designs and statistical approaches. On the other hand, Roshandel and colleagues [96] investigated four epigenetic clocks (DNAmAge, DNAmAgeSkinBloodClock, PhenoAge, and DNAmGrimAge) in relation to complications in type 1 diabetes and confirmed a positive association between DNAmGrimAge and neuropathy. Telomere shortening, another surrogate of biological aging, was also accelerated in diabetic neuropathy [97], although McCartney et al. [98], Horvath et al. [99], and Vetter et al. [100] did not provide support for this hypothesis. Overall, the relationship between NP and biological age could have bidirectional dynamics, with chronic pain being a symptom of aging and a driver of accelerated aging via epigenetic pathways.

## Conclusions

We have comprehensively described alterations in DNA methylation-based clocks, cell count estimates, and surrogates of plasma protein levels in painful and painless DN, and identified significant correlations between these epigenetic biomarkers and neuropathic pain. Our study provides the first evidence that biological age acceleration is associated with the development of pain in diabetic neuropathy. These findings indicate that the aging process may be directly involved in the progression towards the diabetic neuropathy with pain and in general in a health degeneration in T2DM. With that said, it is possible to hypothesize that the administration of effective anti-aging therapeutics could slow down or even block the progression towards neuropathic pain and fitness derangement of the patients. Therefore, presented epigenetic signatures could be useful to better profile patients at risk of developing painful DN and also lead to the development of potential new avenues of treatment.

## Supplementary Information

Below is the link to the electronic supplementary material.

Supplementary file1 (PDF 372 KB)

Supplementary file2 (PDF 367 KB)

Supplementary file3 (PDF 371 KB)

Supplementary file4 (PDF 367 KB)

Supplementary file5 (PDF 367 KB)

Supplementary file6 (PDF 364 KB)

Supplementary file7 (PDF 367 KB)

Supplementary file8 (PDF 370 KB)

Supplementary file9 (PDF 369 KB)

Supplementary file10 (PDF 368 KB)

Supplementary file11 (XLSX 23 KB)

Supplementary file12 (XLSX 11 KB)

Supplementary file13 (XLSX 9 KB)

Supplementary file14 (XLSX 9 KB)

Supplementary file15 (XLSX 8 KB)

Supplementary file16 (XLSX 8 KB)

Supplementary file17 (XLSX 19 KB)

## Data Availability

The datasets generated and analyzed during the current study are available in the GEO NCBI repository under accession number GSE286347.

## Abbreviations

NP: Neuropathic pain
DN: Diabetic neuropathy
T2DM: Type 2 diabetes mellitus
PDN: Painful diabetic neuropathy
PLDN: Painless diabetic neuropathy
CTRL: Healthy controls
