# Supplementary material for: Painful diabetic neuropathy is associated with accelerated epigenetic aging
Source: GeroScience. 2025 Jan 23;47(3):4041–54. doi: 10.1007/s11357-025-01516-w (PMC12181573; doi:10.1007/s11357-025-01516-w)
Supplement: Supplementary file 7 — Supplementary file7 (PDF 367 KB) [file 11357_2025_1516_MOESM7_ESM.pdf]

Article title: Painful diabetic neuropathy is associated with accelerated epigenetic aging
Journal name: GeroScience
Author names: Katarzyna M. Kwiatkowska, Paolo Garagnani, Massimiliano Bonafé, Maria Giulia Bacalini, Luciano Calzari, Davide Gentilini, Dan Ziegler, Monique M. Gerrits, Catharina G. Faber, Rayaz A. Malik, Margherita Marchi, Erika Salvi, Giuseppe Lauria# and Chiara Pirazzini
# Corresponding author: Katarzyna M. Kwiatkowska; Department of Medical and Surgical Sciences (DIMEC), University of Bologna, 40126 Bologna, Italy; katarzyn.kwiatkowsk2@unibo.it
# Corresponding author: Paolo Garagnani; Department of Medical and Surgical Sciences (DIMEC), University of Bologna, 40126 Bologna, Italy; IRCCS Azienda Ospedaliero-Universitaria di Bologna, 40138 Bologna, Italy; paolo.garagnani2@unibo.it

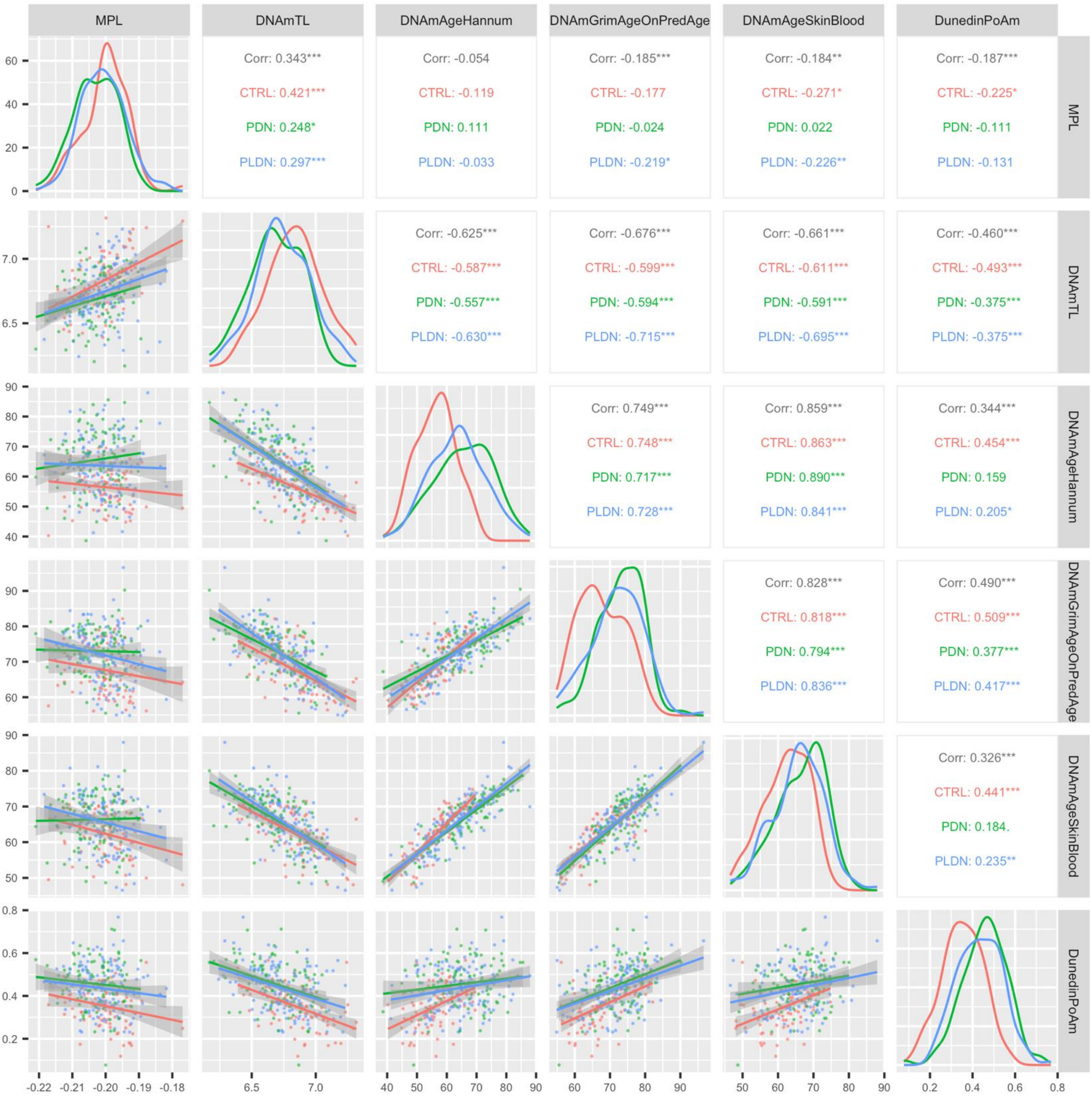

Supplementary Figure S7. Pearson's rank correlation between *MPL* EpiScore and significant epigenetic clocks from subset A. The black, red, green, and blue colors correspond to correlations in entire cohort (n = 315), PDN (n = 99), PLDN (n = 132), and CTRL (n = 84) groups, respectively. Statistical significance is denoted by asterisks, with * representing p-values < 0.05, ** indicating p-values < 0.01, and *** indicating p-values < 0.001.
